# Unexpected observations after mapping LongSAGE tags to the human genome

**DOI:** 10.1186/1471-2105-8-154

**Published:** 2007-05-15

**Authors:** Céline Keime, Marie Sémon, Dominique Mouchiroud, Laurent Duret, Olivier Gandrillon

**Affiliations:** 1 Université de Lyon, Lyon, F-69003, France ; Université Lyon 1, Lyon, F-69003, France, CNRS, UMR5534, Centre de génétique moléculaire et cellulaire, Villeurbanne, F-69622, France; 2 Smurfit Institute of Genetics, Trinity College Dublin, Dublin 2, Ireland; 3 Université de Lyon, Lyon, F-69003, France ; Université Lyon 1, Lyon, F-69003, France, CNRS, UMR5558, Laboratoire de Biométrie et Biologie Evolutive, Villeurbanne, F-69622, France

## Abstract

**Background:**

SAGE has been used widely to study the expression of known transcripts, but much less to annotate new transcribed regions. LongSAGE produces tags that are sufficiently long to be reliably mapped to a whole-genome sequence. Here we used this property to study the position of human LongSAGE tags obtained from all public libraries. We focused mainly on tags that do not map to known transcripts.

**Results:**

Using a published error rate in SAGE libraries, we first removed the tags likely to result from sequencing errors. We then observed that an unexpectedly large number of the remaining tags still did not match the genome sequence. Some of these correspond to parts of human mRNAs, such as polyA tails, junctions between two exons and polymorphic regions of transcripts. Another non-negligible proportion can be attributed to contamination by murine transcripts and to residual sequencing errors. After filtering out our data with these screens to ensure that our dataset is highly reliable, we studied the tags that map once to the genome. 31% of these tags correspond to unannotated transcripts. The others map to known transcribed regions, but many of them (nearly half) are located either in antisense or in new variants of these known transcripts.

**Conclusion:**

We performed a comprehensive study of all publicly available human LongSAGE tags, and carefully verified the reliability of these data. We found the potential origin of many tags that did not match the human genome sequence. The properties of the remaining tags imply that the level of sequencing error may have been under-estimated. The frequency of tags matching once the genome sequence but not in an annotated exon suggests that the human transcriptome is much more complex than shown by the current human genome annotations, with many new splicing variants and antisense transcripts. SAGE data is appropriate to map new transcripts to the genome, as demonstrated by the high rate of cross-validation of the corresponding tags using other methods.

## Background

Serial Analysis of Gene Expression (SAGE) [1] is a widely used method for transcriptome analysis. This technique has been successfully used for the analysis of a variety of biological phenomena, by investigating the expression level of previously characterized mRNAs [2]. It has also permitted the study of important structural characteristics of the human genome such as co-expressed gene clusters [3,4]. More recently, a SAGE library construction pipeline has been described [5], that allows to generate high-quality digital gene expression profiling data.

The SAGE method consists of sequencing small tags derived from the 3' ends of mRNAs. A crucial step in SAGE analysis is tag identification [6,7], or finding the transcript from which each tag was derived. The original SAGE protocol [1] produces 14 bp tags that can be mapped to a set of transcribed sequences with known 3' ends [3,7-9]. Using known transcripts, several studies have shown that 93.4 to 98.5% of human transcripts have unique SAGE tags [9-11]. This proportion is possibly a slight overestimate, because not all human transcripts have yet been annotated. To annotate such new transcripts, it is necessary to directly map the tags to the human genome sequence. However, 14 bp SAGE tags are too short to be reliably mapped only once to the human genome sequence, to the region from which the tag was derived. In contrast, 21 bp tags generated by a modified SAGE protocol called LongSAGE [12] can be identified by mapping them directly to the human genome sequence [12]. Indeed, if we assume a simple model in which the nucleotides are randomly distributed along the genome sequence, and the four bases are equally abundant, each 14 bp tag should map spuriously on average 12 times to the human genome (the probability of matching at least once is *p *= 1 - CN0
 MathType@MTEF@5@5@+=feaafiart1ev1aaatCvAUfKttLearuWrP9MDH5MBPbIqV92AaeXatLxBI9gBaebbnrfifHhDYfgasaacH8akY=wiFfYdH8Gipec8Eeeu0xXdbba9frFj0=OqFfea0dXdd9vqai=hGuQ8kuc9pgc9s8qqaq=dirpe0xb9q8qiLsFr0=vr0=vr0dc8meaabaqaciaacaGaaeqabaqabeGadaaakeaacqWGdbWqdaqhaaWcbaGaemOta4eabaGaeGimaadaaaaa@2FFB@ × (1 - (1/4)^*L*^)^*N *^where *L *= 14 is the tag length and *N *= 3.272.204.263 represents the sum of the lengths of the mitochondrial and nuclear genomes. Therefore, *p *= 0.99). For a LongSAGE tag of 21 bp, this probability of a spurious match is much smaller (*p *= 0.000744). Therefore the LongSAGE tags are much more specific than 14 bp SAGE tags, even if the specificity of LongSAGE tags is not as high as these theoretical calculations suggest, as nucleotides are not randomly and equally distributed along the genome sequence, and the genome contains many repetitive sequences.

A systematic annotation of new transcripts by mapping a library containing 28,000 of these LongSAGE tags to the human genome sequence revealed 15,000 exons that are not currently described, at least half of which belong to novel genes [12]. More recently, this LongSAGE technique has been used to generate mouse libraries, and the analysis of these libraries provides evidence for the existence of about 24,000 previously undescribed transcripts [13]. To find new transcripts, several recent microarray analyses assayed transcription at regular intervals in 10 human chromosomes. They confirmed the existence of a large amount of transcription outside the boundaries of known genes. These new transcripts may double the number of genes compared to current annotations [14-18]. Because these new transcripts tend to be weakly expressed and non-conserved between human and mouse [15,17], it has been argued that they correspond to spurious transcripts. However, transposable elements are excluded from some of these new transcripts, which confirm that some of them are functional [19]. But a comparison of several microarray studies has shown that a rather low percentage of positive probes overlap between experiments, suggesting either a non-negligible false positive rate or a high specificity of different microarray platforms and tissues analyzed [20].

For this reason, we propose to study this question across the whole human genome using an independent method. We exploited the advantages of the LongSAGE method to study the transcriptome without *a priori *knowing the transcribed sequences. We made a comprehensive study of all tags from all publicly available human LongSAGE libraries deposited in the public Gene Expression Omnibus databank. Most of the studies using 14 bp SAGE tags have focused on the expression of known genes. By contrast, here we concentrated on the tags that have not been generated by known transcripts. Because the main difficulty in estimating the amount of transcription in the human genome seems to be the false positive rate of detection [20], we first carefully filtered our dataset and checked the reliability of the remaining tags. After having discarded the tags likely to contain sequencing errors from our dataset, we observed that an unexpectedly large number of tags do not match the genome sequence. We demonstrated that some arise from murine contaminants, polyA tails, junctions between two exons, or polymorphisms. Several arguments lead us to conclude that the remaining tags probably arise from sequencing errors. We estimated therefore that the rate of sequencing error is higher than previously thought. We then studied the tags that map uniquely to the genome, and we showed that 31% of them are located in parts of the genome that are still to be annotated. Among the others, nearly one half correspond to antisense transcripts or to new variants of known transcripts. This shows that the human transcriptome is much more complex than shown by the current genome annotations.

## Results and discussion

### Selection and mapping of reliable tags

We used all the tags available in the public human LongSAGE libraries of the Gene Expression Omnibus database [21]. This corresponds to 29 libraries, generated mainly from stem cell lines or tumoral tissues, but also from several normal tissues (the characteristics of these libraries are provided as supplementary material [see Additional file 1]). By pooling the tags from all these libraries, we obtained a dataset of 3,616,090 tags, corresponding to 630,837 different tags (in other words there are 630,837 unique tags in our dataset). We will hereafter refer to the frequency of tags by comparison to this number of different tags.

#### Tags present only once in the libraries

To be able to predict with sufficient confidence which regions of the genome have generated these SAGE tags, we selected a reliable set of tags from this total dataset. For this purpose, we first considered the tags present only once in our dataset, that have therefore been observed only once in a single SAGE library. Some of these infrequent tags correspond to very weakly expressed transcripts. Others, however, may be incorrect because they have undergone sequencing error(s) during the construction of SAGE libraries. Tags occurring only once represent 13% of the total dataset, and a large proportion (75%) of the different tags. This proportion is not negligible, but as some of these tags, unfortunately, may be incorrect, we checked the reliability of this set of tags before including it in our analysis.

As mentioned above, the probability that a 21 bp sequence spuriously matches the human genome sequence is very small : if the subset of tags occuring only once in the total dataset contained many incorrect tags, it should therefore be enriched in unmapped tags. We therefore mapped each tag to the human nuclear and mitochondrial genome sequence, and compared the tags occurring only once in the SAGE libraries and the tags occurring more than once. Among the subset of tags occurring only once, 73% are unmapped. In contrast, in the other pool of tags, significantly less tags (39%) have not been localized.

Each transcript generates several tags, that could either be correct or incorrect after sequencing: a large majority of these tags are correct, but a small number are incorrect tags containing one or more sequencing errors. For each incorrect tag present in our dataset, it should be possible to recover somewhere else in our dataset the corresponding correct tag, without sequencing error. Thus, for each tag which is present only once and does not match the genome sequence, we checked whether we could find in our dataset another tag matching the genome sequence and identical to this tag apart from one or two base pairs (substitution, insertion or deletion). For 69% of the unmapped tags occurring only once, we found at least one mapped variant. This frequency drops to 33% in the subset of unmapped tags occurring more than once in our dataset.

In conclusion, these results suggest that the subset of tags occurring only once is particularly enriched in incorrect tags resulting from sequencing errors. We have therefore chosen not to include these tags in our analysis.

#### Tags present more than once but due to sequencing error(s)

Excluding tags that are present only once does not eliminate all tags containing sequencing errors. Indeed, the same error could occur several times (especially for tags generated by highly expressed transcripts). We tried therefore to eliminate incorrect tags that occur more than once in the libraries.

For this purpose, we implemented the algorithm proposed by Colinge and Feger [22] (see Methods). To our knowledge, this is the most appropriate method to find possible erroneously sequenced tags in the absence of the corresponding sequence chromatograms. If each tag has the same probability to be erroneous (estimated to 17.3% in LongSAGE libraries [23]), we expect the number of incorrect tags generated by a transcript to be proportional to the total number of tags generated by this transcript. Then, given the number of occurrences of a given tag *t*, we can evaluate the number of variants derived from this tag *t *by sequencing errors. We identified the set of tags corresponding to all the variants of *t *(differing by at most two base pairs because of substitution(s), insertion(s) or deletion(s)), and determined for each variant whether it was rare enough to be only due to sequencing error(s). If so, the variant was discarded from the dataset.

Ultimately, by eliminating tags present only once and tags occurring more than once but probably erroneous, we removed on average 17.46% of the tags per library. After this filtering step, our set of reliable tags contained 3,115,752 tags, corresponding in total to 148,553 different tags.

#### Mapping the tags to the genome

**Figure 1:**
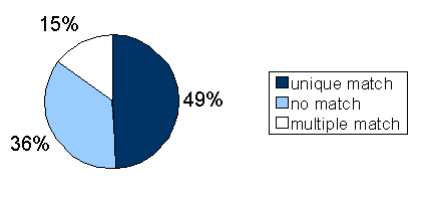
**Localization of the LongSAGE tags in the human genome**. Results of the mapping of the reliable tags from all publicly available human LongSAGE libraries (148,553 tags) to the human nuclear and mitochondrial genomes. Only matches with 100% similarity over 21 bp are included in the dataset.

Figure 1 shows the results obtained after mapping all tags from our set of reliable tags to the human nuclear and mitochondrial genomes (further details on these results are provided as supplementary material [see Additional file 2]). Nearly half of the set of reliable tags map once to the human genome. Tags that do not fulfill this expectation correspond either to tags found at multiple positions in the genome (15%) or to unmapped tags (36%). The latter are surprisingly numerous. A similar analysis using 14 bp SAGE data led to drastically different results, as 97% of these tags mapped to several locations in the human genome sequence (data not shown). This confirms the advantage of the LongSAGE method over the original SAGE method.

Saha *et al *have already shown that most of the LongSAGE tags that match several positions in the genome sequence correspond to duplicated genes or tandem repeats [12]. Using tags from chicken LongSAGE libraries constructed in our laboratory (Keime et al., in preparation), we observed that the fraction of chicken tags that match several positions in the chicken genome sequence is much smaller (7% of the chicken tags that match the chicken genome sequence map to several locations in the chicken genome, data not shown) than human tags in the human genome (23% of the human tags that match the human genome sequence map to several locations in the human genome). This is consistent with observations showing that the frequency of repeated sequences and duplicated genes is smaller in the chicken than in the human genome [24].

We will now focus on the two remaining sets in Figure 1, unmapped tags and tags that map once to the genome. Each of them raises a specific question. What is the origin of the unmapped tags? And do all uniquely mapped tags correspond to annotated transcripts?

### Analysis of tags that do not map to the genome

**Figure 2:**
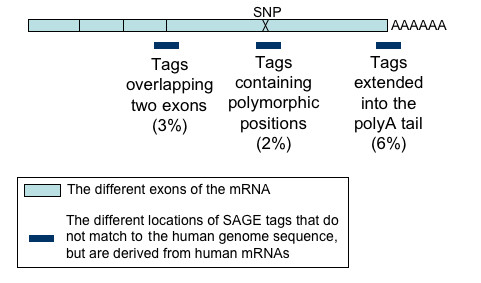
**Tags that do not match the human genome sequence, but are derived from human mRNAs**. This figure shows different situations which lead to tags that do not match the human genome sequence, even if they are derived from human mRNAs. The expected proportion of such tags among all different tags (calculated using known transcript sequences) are shown in brackets.

Because we tried to remove tags generated by sequencing errors, we expected to see only very few unmapped tags, and not 53551 tags, which corresponds to 36% of the total set of different tags. We therefore tried to understand why these tags were present in the LongSAGE libraries. We first checked different possibilities that would explain why several tags generated by human mRNAs do not map to the genome, for instance, tags overlapping two exons, tags extended into the polyA tail and tags that differ from the genome sequence because of polymorphism. The results, presented below, are summarized in Figure 2.

#### Tags overlapping two exons

Tags that do not map to the genome could correspond to tags overlapping two exons. We computed the expected proportion of such tags using a set of transcripts with reliably annotated exons. For this purpose, we extracted *in silico *tags from 20281 transcripts annotated in Refseq (and containing both a polyadenylation signal and a NlaIII restriction site). In these sequences, 3% of the tags overlap two exons.

Among the tags from our dataset that do not map to the genome, we found that 1885 different tags overlap two exons, by using Ensembl annotations. These tags correspond to 1% of our initial set of tags. This proportion is slightly lower than the expected value, no doubt because the quality of annotations for all transcripts is not as high as in the set of Refseq transcripts.

#### Tags extended into the polyA tails

Tags containing part of the polyA tail can also not be mapped to the genome sequence. We computed the expected proportion of such tags using a set of transcripts for which the polyA tail is known. For this, we extracted *in silico *the LongSAGE tag from 12418 Refseq transcripts (manually annotated and containing a polyA tail defined by at least 10 "A" bases downstream of a polyadenylation signal present in the last 50 bases of the sequence) : 6% of these tags extend into the polyA tail.

To estimate the frequency of such tags in our dataset, we mapped these tags to all human ESTs available in dbEST [25]. We also considered each tag ending in base "A", because they may extend into the polyA tail. For each of these tags, we extracted the set of EST sequences containing this tag, and then we trimmed these sequences to obtain the parts downstream of the tag. We then computed the frequency of "A" in these fragments, and considered the tag to extend into the polyA tail if this frequency exceeded 70% in at least one of these fragments. Using this method, we found that 1170 tags unmapped to the genome extend into polyA tails, which corresponds to 0.8% of our initial set of tags. This proportion is lower than the expected value (6%), almost certainly because not all unmapped tags could be mapped to an EST sequence and the polyA tail is not sequenced on the 3' end of every EST sequence. However, as the position of the polyA tail is not exactly the same in each mRNA corresponding to the same gene, the Refseq and EST sequences only represent one possibility for the position of the polyA tail. Therefore, our estimates of the expected and observed proportions of the tags that contain a polyA tail are likely to be underestimates.

#### Tags containing polymorphic positions

Unmapped tags may also be due to the presence of a polymorphic region of the genome (Single Nucleotide Polymorphism : SNP), if the allele sequenced in the genome project differs from the allele of the individual used to construct the SAGE library. It has previously been estimated that any two copies of the human genome differ from one another by approximately 0.1% of nucleotide sites (that is, one variant per 1,000 bases on average) [26]. Therefore, the probability *p *that a given tag contains no SNP is *p *= (1 - 1/1000)^21^, and the expected proportion of tags with at least one polymorphic site is roughly 2% (1 - *p*).

We searched for the presence of such tags among our set of unmapped tags. For this purpose, we used a dataset computed using a previously published method [27] (Anamaria Camargo, personal communication): UniGene cluster sequences were searched for the presence of SNPs (according to the NCBI SNP Database), either within the tag sequence or within the restriction enzyme site used for SAGE library construction. By using this dataset, we found that 213 tags from our set of unmapped tags could be due to the presence of a polymorphic region of the genome. These tags correspond to 0.1% of our initial set of tags. The observed frequency is therefore lower than the expected one. However, the expected frequency was calculated using the frequency of SNPs estimated using known sequences. We expect that this frequency would be lower for sequences that are not well characterized yet. This could partly explain why we observe fewer SNPs than the theoretical value we calculated. Furthermore, the observed frequency of SNPs is certainly lower than the real value. Indeed, SNP alternative tags could only reliably be predicted on complete mRNA (with a polyA tail) that could be mapped to the human genome [27].

#### Tags belonging to EST sequences

These explanations are not entirely satisfactory, because we expect only 10% of all different tags to correspond to any of the cases mentioned above, but we observe that 36% of all tags do not match the genome sequence (see Figure 1). Our theoretical calculations rely on the quality of the annotations and could thus possibly underestimate the real values. Therefore, we tried to map each unmapped tag to human EST sequences. 15424 unmapped tags match at least one EST, which represents 10% of the total of the set of different tags (or 29% of the set of unmapped tags). This result is in agreement with our theoretical expectations. In conclusion, we did not find the potential origin of all unmapped tags : it seems that some fraction of these tags do not correspond to the sequence of already known human mRNAs.

#### Tags generated by contaminants

**Figure 3:**
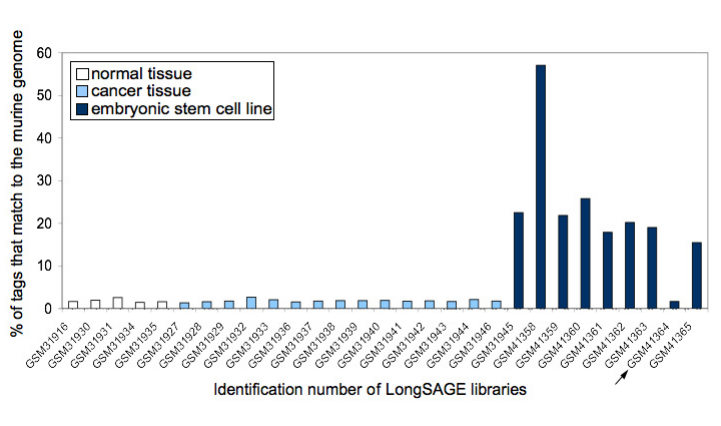
**Proportion of tags mapping to the murine genome**. In order to search for probable murine contaminants among our set of tags, we mapped to the murine genome all tags that do not match the human genome sequence and for which we could not find any other human origin. This figure shows the percentage of these tags that could be localized on the murine nuclear and mitochondrial genome, for each public LongSAGE library. The identification number of the LongSAGE libraries in the Gene Expression Omnibus repository is indicated on the x axis. All embryonic stem cells were propagated on murine embryonic fibroblasts excepted the ones used to construct the library indicated by an arrow.

One third of the human public LongSAGE libraries have been obtained from embryonic stem cell lines, which are often propagated on mouse embryonic fibroblasts (MEF) [28]. Some MEF may therefore have been included in the embryonic stem cell preparation, and murine mRNA may thus have contaminated the SAGE libraries. We expect that such murine tags would not match the human genome sequence in many cases. Indeed, only 6% of murine virtual LongSAGE tags we extracted from 6160 Refseq sequences match the human genome sequence. We thus looked for mouse tags in the set of tags that do not match the human genome sequence and that do not correspond to any of the cases previously described. For this purpose, we mapped these tags to the mouse nuclear and mitochondrial genomes. Figure 3 shows that the proportion of tags that map to the mouse genome is higher in the libraries that have been obtained from embryonic stem cells than from other tissues. Among the nine cell populations used to construct these libraries, eight have been propagated on murine fibroblasts (according to library annotations or to the submitter of the corresponding SAGE libraries : Meri Firpo, Daniela S. Gerhard and Martin Pera, personal communication). The remaining library, indicated by an arrow on Figure 3, was constructed using mRNA from embryonic stem cells which were not propagated on murine fibroblasts. This probably explains why the frequency of tags mapping to the murine genome in this library is not higher than the frequency in libraries obtained from other tissues (Man Whitney p = 0.64).

We considered a tag as a contaminant if it did not map to the human genome, nor to a human mature transcript, it occured only in embryonic stem cell libraries propagated on MEF, and it mapped to the mouse genome. These tags represent a non-negligible proportion (13%) of the tags that do not match the human genome sequence.

It is obvious that the percentage of tags that map to the mouse genome varies between embryonic stem cell libraries, revealing different degrees of exclusion of MEF. Our results show that even when libraries have been constructed from carefully dissected material, it is always necessary to filter the tags to exclude tags generated by transcripts present in the remaining MEF.

#### Where do the remaining tags come from ?

In total, the origin of 42% of the unmapped tags was explained by one of the situations previously described (29% correspond to a human transcript and 13% correspond to mouse contaminants). However, we could not explain the origin of the remaining unmapped tags. These tags do not belong to any library in particular. The large majority (91%) of these tags correspond to sequences varying by one base from another tag that maps to the genome, and some of these tags could therefore correspond to rare polymorphisms that are not represented by an EST. This is possible, but unlikely to be the main explanation because we studied twice as many ESTs (6 × 10^6^) as SAGE tags (3.1 × 10^6^). We also tested whether these tags could come from edited mRNAs that are not represented among EST sequences. For this purpose, we examined the transition frequencies when comparing genomic and tag sequences, since the two known families of RNA-editing enzymes in humans perform adenosine to inosine or cytosine to uracil modifications [29]. However, these modifications are not overrepresented in our dataset [see Additional file 3]. Therefore, the set of unmapped tags for which we could not find any origin seem do not seem to be enriched in tags coming from A-to-I or C-to-U edited mRNA.

#### Necessity of reassessing the error rate in SAGE libraries

Unmapped tags whose origin could not be explained by our previous screens occur on average at a low frequency : 88% occur 5 times or less in the dataset, and the vast majority of these tags correspond to sequences varying by one base from another tag that maps to the genome (see above). Because of this, and because our screens exclude many other possible explanations, we think that the most parsimonious explanation for the presence of these tags is that they contain sequencing error(s). We initially used an error rate that was previously published (17.3% of LongSAGE tags contain at least one error [23]) to remove the tags containing sequencing error(s). The observation of many unmapped tags that are likely to contain errors suggests that this error rate needs to be reevaluated. In the different libraries we analyzed, the unmapped tags whose origin could not be explained by our previous screens represent from 1 to 8% of of the size of the library (total number of tags). Therefore, we used the same method as we initially used to remove sequencing errors (see methods), but with an higher error rate (17.3 + 8 = 25.3%). With this new filter for sequencing errors, the set of unexplained tags drops dramatically (less than 1% per library on average), suggesting that the majority of the tags whose origin could not be explained by our previous screens are probably due to sequencing errors.

### Analysis of tags that map to only one location on the genome

Tags mapping once to the human genome represent nearly half (49%) of the set of different tags. We studied the localization of these tags with respect to known transcripts, to evaluate the amount of transcription inside and outside annotated transcripts.

#### Tags mapping to annotated transcripts

We first studied the tags that are located inside known transcripts, using Ensembl annotations [30]. These annotations do not always provide the complete 3'UTR (UnTranslated Region). Indeed, the annotation of these regions is particularly difficult because it relies on the availability of a cDNA sequence complete in 3' ([31,32]). We have therefore extended each transcript by systematically adding 500 bp to the annotated 3'UTR. We chose this threshold because it adjusts the average length of the annotated UTRs in Ensembl (641 bp in our dataset) to the average length of human 3'UTR in UTRdb (1,400 bp in a dataset containing 4,845 human 3'UTR [33]).

**Figure 4:**
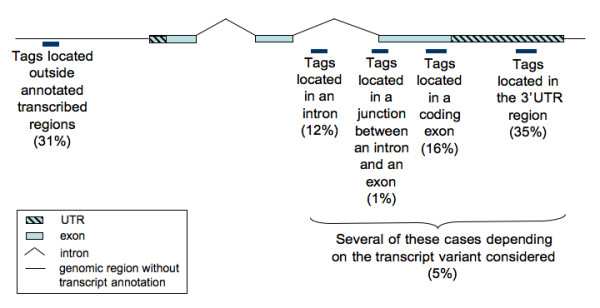
**Tags that match uniquely the human genome sequence**. Proportion of tags found in different genic regions among the tags that match uniquely the human genome sequence.

Among the tags mapping once to the genome, 69% are located in such "extended" transcripts. A more precise description of the tag positions in different parts of these transcripts is displayed in Figure 4. As expected, a large proportion of the tags map to the 3'UTRs, and most of the others map to the coding part of exons. Many tags map both to an intron and to an exon, depending on the splicing variant considered. After discarding these cases, 12% of the tags matching once the genome sequence map to an intron (and 1% are located in a junction between an intron and an exon). These tags do not belong to an annotated mature mRNA, and therefore they correspond either to new splicing variants of known genes or to new transcripts that overlap with known transcripts.

Even among the tags mapping to annotated transcripts, a non-negligible proportion (32%) maps in antisense compared to the annotated transcribed strand. Such tags have already been highlighted by several previous studies [34-36]. We observed that the proportion of tags mapping to antisense is significantly higher for tags located in 3'UTR (36.6%) than for tags located in coding exons (25.2%, chi-squared test, *p *< 10^-16^). This means that a large proportion of the genome is transcribed from both strands of the DNA, especially in the 3'end of the transcripts, confirming previous expectations [20]. Among the 19,800 transcripts for which we found at least one tag in out dataset, 36.1% possess a tag in antisense. This proportion is in accordance with observations in *Arabidopsis thaliana *[37], but is higher than previous observations in human (between 5% and 20% of all genes studied were found to have an antisense counterpart, in the different studies already published. For a review of these studies see [38]). For each of the public LongSAGE libraries, we computed the proportion of transcripts with a tag in the same orientation as the annotated one, in the opposite orientation, or in both orientations. We found that on average 61% of the transcripts per library have tags only in the same orientation as the annotated one, 10% have tags only in the antisense orientation, and 29% have tags in both senses. However, these proportions varied between libraries, notably with library size (the more the library has been sequenced, the higher the proportion of transcripts for which we found corresponding tags both in sense and antisense orientation). This could be explained by the low abundance of antisense transcripts, which could only be detected by in-depth sequencing. Indeed, for the transcripts with corresponding tags in both orientation, tags in the same orientation as the annotated one are usually more abundant than tags in antisense (this in the case for 69% of the transcripts, on average across the different libraries).

#### Tags mapping outside annotated transcripts

31% of tags mapping once to the genome (which correspond to 22,441 tags) do not correspond to a known transcript. We already have discarded the tags that could have been generated by sequencing errors using a published sequencing error rate in SAGE libraries. However, as we previously mentioned, it is possible that the error rate is higher than anticipated, and that some tags from our dataset still contain sequencing errors. We thus estimated the probability that such tags containing a sequencing error match the human genome sequence. For this purpose, we randomly selected 100,000 tags that match the human genome sequence, and we modified them by introducing "sequencing errors". These errors were randomly attributed, by using the percentage of error for each base we calculated (see methods). We found that 7.6% of these modified tags matched the human genome sequence : this is an estimate of the probability that an erroneous tag maps to the genome (*p*(*match*|*erroneous*)). Consequently, for each tag from our dataset that maps to the genome (once or more than once), the probability that it is erroneous can be estimated by the following calculation :

p(erroneous|match)=p(match|erroneous)×p(erroneous)p(match)=0.076×p(erroneous)0.64
 MathType@MTEF@5@5@+=feaafiart1ev1aaatCvAUfKttLearuWrP9MDH5MBPbIqV92AaeXatLxBI9gBaebbnrfifHhDYfgasaacH8akY=wiFfYdH8Gipec8Eeeu0xXdbba9frFj0=OqFfea0dXdd9vqai=hGuQ8kuc9pgc9s8qqaq=dirpe0xb9q8qiLsFr0=vr0=vr0dc8meaabaqaciaacaGaaeqabaqabeGadaaakeaacqWGWbaCcqGGOaakcqWGLbqzcqWGYbGCcqWGYbGCcqWGVbWBcqWGUbGBcqWGLbqzcqWGVbWBcqWG1bqDcqWGZbWCcqGG8baFcqWGTbqBcqWGHbqycqWG0baDcqWGJbWycqWGObaAcqGGPaqkcqGH9aqpdaWcaaqaaiabdchaWjabcIcaOiabd2gaTjabdggaHjabdsha0jabdogaJjabdIgaOjabcYha8jabdwgaLjabdkhaYjabdkhaYjabd+gaVjabd6gaUjabdwgaLjabd+gaVjabdwha1jabdohaZjabcMcaPiabgEna0kabdchaWjabcIcaOiabdwgaLjabdkhaYjabdkhaYjabd+gaVjabd6gaUjabdwgaLjabd+gaVjabdwha1jabdohaZjabcMcaPaqaaiabdchaWjabcIcaOiabd2gaTjabdggaHjabdsha0jabdogaJjabdIgaOjabcMcaPaaacqGH9aqpdaWcaaqaaiabicdaWiabc6caUiabicdaWiabiEda3iabiAda2iabgEna0kabdchaWjabcIcaOiabdwgaLjabdkhaYjabdkhaYjabd+gaVjabd6gaUjabdwgaLjabd+gaVjabdwha1jabdohaZjabcMcaPaqaaiabicdaWiabc6caUiabiAda2iabisda0aaaaaa@94AE@

With a sequencing error rate *p*(*erroneous*) = 0.173, *p*(*erroneous*|*match*) = 0.020, and even with a higher error rate (*p*(*erroneous*) = 0.253), *p*(*erroneous*|*match*) = 0.030 is still low.

Because the probability that a mapped tag is erroneous is very small, the majority of the tags mapping once to the genome and outside annotated transcripts should come from unknown transcribed regions. However, it is possible that some of these tags do not belong to new transcripts, because the real 3'UTR may be longer than annotated (even after our extension). We therefore calculated the distance between these tags and the 3' end of the nearest transcript. We observed that very few tags are located in an incompletely annotated 3'UTR (less than 2% of tags that do not correspond to a known transcript are closer than 1000 bp to the nearest transcript).

Because the vast majority of tags that do not correspond to an annotated transcript do not originate from an incompletely annotated 3'UTR, we searched for other evidence of transcription in the regions from which these tags originate (see Table 1 and text below).

**Table 1:** Distribution of tags that match once the genome sequence outside annotated transcripts

	Number	% of tags matching outside annotated transcripts
Transposable element	2874	12
EST	18042	78
Transfrag	2017	9
EST transfrag	18180	79
Transposable element EST transfrag	21054	83

Number of tags outside annotated transcripts. The percentage values correspond to the proportion of each of these sets of tags compared to the set of all tags matching once in the genome, outside an annotated transcript.

We found that 12% of these tags are located in a transposable element (for this purpose, we annotated transposable elements in the 4,000 bp surrounding each tag using RepeatMasker [39]). We also found that 78% of the tags that match once the genome sequence but not in a known transcript map to at least one human EST (from dbEST). This confirms, using independent evidence, that these tags mapping outside annotated transcripts belong to real transcripts.

As we mentioned in the introduction, most of the recent work on finding new transcripts in the human genome has been performed using tiling microarrays. We thus compared our SAGE tags (mapping once to the genome but not on known transcripts) with transcribed regions predicted using tiling microarrays. For this comparison, we used the transfrags (transcribed fragments, [18]) recently obtained by Cheng et al. by studying transcribed sequences, polyadenylated or not, from ten human chromosomes (that represent approximately 30% of the human genome). 35% of our set of tags not located on an annotated transcript and that are located on the 10 chromosomes studied by Chen et al. map to such a transfrag (this represents 9% of the total set of tags not located on an annotated transcript). For these tags, we thus have two independent lines of evidence that they come from a transcribed region.

Conversely, only 0.39% of the transfrags contain one or more of our tags. We propose several explanations for this observation. Nearly half of the transfrags correspond to nonpolyadenylated transcripts [18] that are not analyzed by SAGE. Some of the transfrags may correspond to transcripts specifically expressed in particular conditions, that have not yet been analyzed by SAGE (note that this should also decrease the percentage of tags that belong to a transfrag). Finally, the small overlap between the results obtained in the different studies using human tiling microarrays suggest either that the transcriptome of the various tissues analyzed is very different, or that these array experiments provide a large fraction of false positives (these two explanations could both be correct) [20]. The fraction of false positives may thus be high, and both the high percentage of our tags that are located on transfrags and the low percentage of transfrags that contain a tag suggest that our set of tags may contain fewer false positives than existing transfrags do.

## Conclusion

Using the SAGE method, it is possible to study the transcriptome without any *a priori *knowledge of expressed genes. We used all the human LongSAGE libraries available, filtered them to remove tags containing sequencing errors, and systematically mapped these tags to the genome. We particularly concentrated on unexpected localizations, either because the tags did not match the genome sequence, or because they mapped outside known transcripts. We then proposed explanations or hypotheses for the origin of these tags.

More than one third of the different tags do not map to the human genome. Among them, 42% are part of mRNA sequences but are not found on the human genome because they correspond to polyA tails, junctions between exons, polymorphic sites or contaminant murine transcripts. The other tags are probably due to sequencing error(s). Consequently, the sequencing error rate in these public libraries is probably higher than previously estimated.

Half of the different tags map once to the genome, and one quarter of these tags match outside annotated transcripts. This suggests that many transcripts are still to be annotated in the human genome. Because many tags mapping to known transcripts belong either to introns or are aligned in antisense, we suggest that they belong to new variants or antisense mRNAs of these transcripts. Consequently, the human transcriptome seems to be more complex than shown by the current genome annotations, and LongSAGE analysis should help to improve the annotation process.

## Methods

### Datasets

SAGE libraries were downloaded from the NCBI website at the following address: , in July 2005.

### Tag mapping

The tags were localized on the human nuclear genome (Ensembl release 24 – NCBI34, October 2004) and mitochondrial genome (Refseq sequence NC_001807) using Megablast [40]. Only matches with 100% identity to the whole length of the tags were accepted. A similar method was used to map the tags to the mRNA sequences.

### Discarding tags generated by sequencing errors

To discard tags that are likely to have been generated by sequencing errors, we implemented Colinge and Feger's method [22]. This method is based on the hypothesis that each tag has the same probability to contain an error. We therefore expect that the number of tags with errors generated by a transcript is proportional to the total number of tags generated by this transcript.

First, it is necessary to know the probability for one particular tag to be sequenced with an error. It has previously been estimated that the error rate is 17.3% in LongSAGE libraries [23]. This represents the probability of finding at least one error in a LongSAGE tag. Under the hypothesis that all errors are independent, we can therefore deduce *x*, the error rate per base: 0.173 = 1 - (1 - *x*)^17^. We obtain *x *= 0.0111, and therefore the probabilities to find exactly one error in one tag (*p*_1 _= 17*x*(1 - *x*)^16^), and exactly two errors in the same tag (*p*_2 _= C172
 MathType@MTEF@5@5@+=feaafiart1ev1aaatCvAUfKttLearuWrP9MDH5MBPbIqV92AaeXatLxBI9gBaebbnrfifHhDYfgasaacH8akY=wiFfYdH8Gipec8Eeeu0xXdbba9frFj0=OqFfea0dXdd9vqai=hGuQ8kuc9pgc9s8qqaq=dirpe0xb9q8qiLsFr0=vr0=vr0dc8meaabaqaciaacaGaaeqabaqabeGadaaakeaacqWGdbWqdaqhaaWcbaGaeGymaeJaeG4naCdabaGaeGOmaidaaaaa@30C6@*x*^2^(1 - *x*)^15^). The probability of finding one or two errors (*p*_1 _+ *p*_2 _= 17.21%) is much greater than the probability of finding more than two errors (*p*_3 _= 0.083%). We therefore ignore *p*_3_.

Let *L *be the set of tags in a given library. We can define for each tag *t *∈ *L *one set of tags *V*_1_(*t*) that contain the tags *q *∈ *L *that can be obtained by changing one base of the tag t (insertion, substitution, or deletion). Likewise, we define *V*_2_(*t*) as the set of tags *q *∈ *L *that vary from t by two changes. As proposed by [22], we computed for each tag *t *the average contribution of its neighbors *q *to the number of occurrences of the tag t. This contribution, *ν*(*t*), can be calculated using the following equation:

ν(t)=∑q∈V1(t)p1×occ(q)#V1(q)+∑q∈V2(t)p2×occ(q)#V2(q)
 MathType@MTEF@5@5@+=feaafiart1ev1aaatCvAUfKttLearuWrP9MDH5MBPbIqV92AaeXatLxBI9gBaebbnrfifHhDYfgasaacH8akY=wiFfYdH8Gipec8Eeeu0xXdbba9frFj0=OqFfea0dXdd9vqai=hGuQ8kuc9pgc9s8qqaq=dirpe0xb9q8qiLsFr0=vr0=vr0dc8meaabaqaciaacaGaaeqabaqabeGadaaakeaaiiGacqWF9oGBcqGGOaakcqWG0baDcqGGPaqkcqGH9aqpdaaeqbqaamaalaaabaGaemiCaa3aaSbaaSqaaiabigdaXaqabaGccqGHxdaTcqWGVbWBcqWGJbWycqWGJbWycqGGOaakcqWGXbqCcqGGPaqkaeaacqGGJaWicqWGwbGvdaWgaaWcbaGaeGymaedabeaakiabcIcaOiabdghaXjabcMcaPaaaaSqaaiabdghaXjabgIGiolabdAfawnaaBaaameaacqaIXaqmaeqaaSGaeiikaGIaemiDaqNaeiykaKcabeqdcqGHris5aOGaey4kaSYaaabuaeaadaWcaaqaaiabdchaWnaaBaaaleaacqaIYaGmaeqaaOGaey41aqRaem4Ba8Maem4yamMaem4yamMaeiikaGIaemyCaeNaeiykaKcabaGaei4iamIaemOvay1aaSbaaSqaaiabikdaYaqabaGccqGGOaakcqWGXbqCcqGGPaqkaaaaleaacqWGXbqCcqGHiiIZcqWGwbGvdaWgaaadbaGaeGOmaidabeaaliabcIcaOiabdsha0jabcMcaPaqab0GaeyyeIuoaaaa@6CE6@

where *occ*(*q*) is the number of occurrences of the tag *q*, and #*V*_*i*_(*q*) the cardinality of *V*_*i*_(*q*).

Therefore, *p*_*i*_/#*V*_*i*_(*q*) corresponds to the average contribution of *q *to each of its neighbors. In other words, each tag will equally contribute to each of its neighbors to increase their number of occurrences.

In each SAGE library, we eliminated all the tags for which *ν*(*t*) ≥ *occ*(*t*), because these tags may be due to sequencing errors.

#### Probability that a tag containing one sequencing error still matches the genome sequence

If we have not discarded all tags containing sequencing error(s), it is possible that some tags containing sequencing error(s) match the genome sequence. We therefore measured by simulation the probability that these tags containing sequencing error(s) map to the genome.

For this purpose, we picked up tags that map once to the genome, and we modified them by introducing "sequencing errors". To obtain tags that could have plausibly been created by sequencing errors, we need to know the probability of finding each base instead of each given base, because of sequencing error (e.g. A changed to a T). By comparing each correct tag (matching once the genome sequence) with incorrect variants of this tag (unmapped to the genome, and containing one or two errors by comparison to the corresponding correct tag), we obtained a matrix with the relative frequencies of each of the 12 sequencing errors (plus the frequencies of deletions and insertions). We then applied one modification per tag, according to this matrix. Then, we checked whether these modified tags mapped to the genome. Finally, we obtained an estimate of the frequency of tags with a sequencing error that map to the genome.

## Authors' contributions

CK and MS designed and performed this study, and wrote the manuscript. LD, OG and DM provided guidance with comments on the study and on the manuscript. All authors read and approved the final manuscript.

## Supplementary Material

Additional File 1 **Characteristics of the LongSAGE libraries analyzed**. Characteristics of the LongSAGE libraries analyzed : their identification number in the Gene Expression Omnibus database (GSM), their total number of tags and their title as provided in the Gene Expression Omnibus database. Click here for file

Additional File 2 **Different origins of LongSAGE tags**. Classification of the different tags by our filtering process. Click here for file

Additional File 3 **Frequencies of base changes between a mapped LongSAGE tag and its variants**. Since the vast majority of the unmapped tags whose origin could not be explained correspond to sequences varying by one base from another tag that maps to the genome, we investigated if these tags could come from edited mRNA. There are two known families of RNA-editing enzymes in human : the adenosine deaminases acting on RNA (ADAR) which perform adenosine-to-inosine (A-to-I) modifications, and the apoB mRNA-editing catalytic peptide (APOBEC) which induces cytosine to uracile (C-to-U) transformations [29]. In human, the most prevalent type of RNA editing is A-to-I [41]. Recent bioinformatic studies have suggested the presence of more than 12,000 A-to-I editing sites corresponding to more than 1,400 edited mRNAs [42-44]. These sites correspond primarily to non-coding regions of the RNA, typically Alu repeats [42-44]. A hallmark of an A-to-I RNA editing event is an A-to-G transition when comparing genomic and cDNA sequences of the affected gene, since inosine base pairs with cytosine and therefore is replaced by guanosine during reverse transcription [42]. We therefore checked whether tags from our set of unmapped tags for which we could not find any origin could come from A-to-I or C-to-U edited mRNA. For this purpose, we built a matrix containing the transition frequencies between base pairs, by comparing correct tags that match with 100% identity to the genome sequence and their corresponding incorrect variants (unmapped to the genome, and containing one modification by comparison to the corresponding correct tag : these variants could either be due to sequencing errors or to editing). This table shows this matrix, where the rows correspond to the bases in the tags mapped to the genome sequence, and the columns the bases in the corresponding variant tags. We see that the transition frequency from A to G is roughly the same as the transition frequency from G to A. Likewise, the transition frequencies from C to T and from T to C are roughly equal. Therefore, the set of unmapped tags for which we could not find any origin do not seem to be enriched in tags coming from A-to-I or C-to-U edited mRNA. Click here for file
