# Supplementary material for: Unexpected observations after mapping LongSAGE tags to the human genome
Source: BMC Bioinformatics. 2007 May 15;8:154. doi: 10.1186/1471-2105-8-154 (PMC1884178; doi:10.1186/1471-2105-8-154)
Supplement: Additional File 1 — Characteristics of the LongSAGE libraries analyzed. Characteristics of the LongSAGE libraries analyzed : their identification number in the Gene Expression Omnibus database (GSM), their total number of tags and their title as provided in the Gene Expression Omnibus database. [file 1471-2105-8-154-S1.pdf]

GSM	Total tag number	Title
31916	21520	LSAGE_Pancreas_normal_B_1
31927	65091	LSAGE_Breast_carcinoma_associated_myofibroblast_AP_IDC7
31928	73410	LSAGE_Breast_carcinoma_epithelium_AP_IDC7
31929	68024	LSAGE_Breast_carcinoma_associated_stroma_B_IDC7
31930	69006	LSAGE_Breast_normal_myoepithelium_AP_IDC7
31931	41773	LSAGE_Brain_normal_substantia_nigra_B_1
31932	21840	LSAGE_Breast_carcinoma_B_CT15
31933	57049	LSAGE_Breast_carcinoma_associated_stroma_B_DCIS6
31934	77759	LSAGE_Vascular_endothelium_normal_liver_associated_AP_NLEC1
31935	305546	LSAGE_Brain_fetal_normal_B_S1
31936	50701	LSAGE_Breast_carcinoma_B_BWHT18
31937	34399	LSAGE_White_Blood_Cells_breast_carcinoma_associated_AP_IDC7
31938	66979	LSAGE_Breast_carcinoma_epithelium_AP_T392303
31939	74264	LSAGE_Breast_carcinoma_associated_myofibroblast_AP_T392303
31940	66299	LSAGE_Breast_carcinoma_epithelium_AP_T112603
31941	63795	LSAGE_Breast_carcinoma_associated_myofibroblast_AP_T112603
31942	59763	LSAGE_Breast_phyllodes_tumor_fibroblasts_AP_070202
31943	68007	LSAGE_Breast_carcinoma_MD_LCIS
31944	55868	LSAGE_Breast_fibroadenoma_MD
31945	401432	LSAGE_Embryonic_stem_cells_H9_normal_p38_CL_SHES2
31946	36321	LSAGE_Lung_adenocarcinoma_B_1
41358	224488	LSAGE_Embryonic_stem_cell_HSF6_normal_p50_CL_SHES9
41359	205353	LSAGE_Embryonic_stem_cell_HES3_normal_p16_CL_SHE10
41360	272465	LSAGE_Embryonic_stem_cell_H7_normal_p33_CL_SHE13
41361	212170	LSAGE_Embryonic_stem_cell_H14_normal_p22_CL_SHE14
41362	209232	LSAGE_Embryonic_stem_cell_HES4_normal_p36_CL_SHE11
41363	221101	LSAGE_Embryonic_stem_cell_H13_normal_p22_CL_SHE15
41364	218214	LSAGE_Embryonic_stem_cell_H1_normal_p54_CL_SHE16
41365	276203	LSAGE_Embryonic_stem_cell_H1_normal_p31_CL_SHE17
