# Supplementary figures and images for: Unexpected observations after mapping LongSAGE tags to the human genome
Source: BMC Bioinformatics. 2007 May 15;8:154. doi: 10.1186/1471-2105-8-154 (PMC1884178; doi:10.1186/1471-2105-8-154)

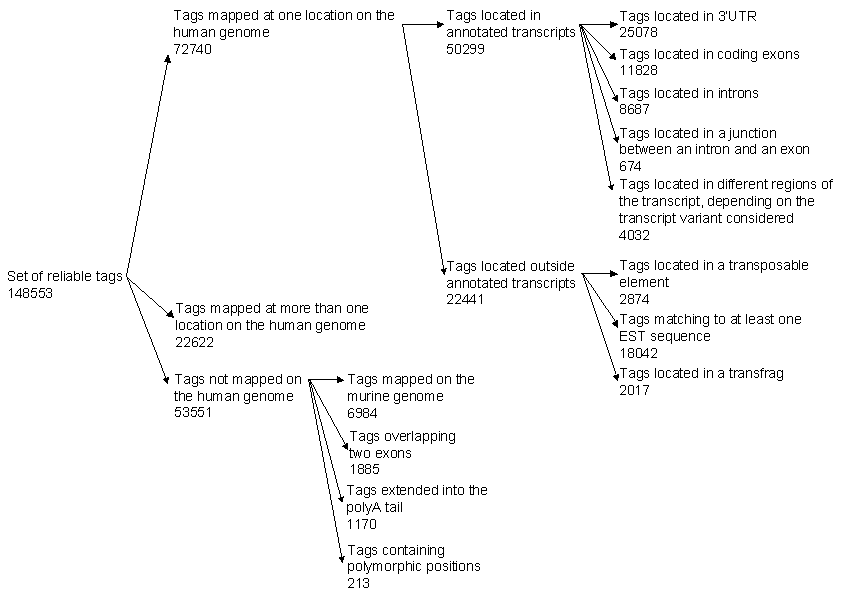

Supplement: Additional File 2 — Different origins of LongSAGE tags. Classification of the different tags by our filtering process. [file 1471-2105-8-154-S2.png]
