# Supplementary material for: Unexpected observations after mapping LongSAGE tags to the human genome
Source: BMC Bioinformatics. 2007 May 15;8:154. doi: 10.1186/1471-2105-8-154 (PMC1884178; doi:10.1186/1471-2105-8-154)
Supplement: Additional File 3 — Frequencies of base changes between a mapped LongSAGE tag and its variants. Since the vast majority of the unmapped tags whose origin could not be explained correspond to sequences varying by one base from another tag that maps to the genome, we investigated if these tags could come from edited mRNA. There are two known families of RNA-editing enzymes in human : the adenosine deaminases acting on RNA (ADAR) which perform adenosine-to-inosine (A-to-I) modifications, and the apoB mRNA-editing catalytic peptide (APOBEC) which induces cytosine to uracile (C-to-U) transformations [29]. In human, the most prevalent type of RNA editing is A-to-I [41]. Recent bioinformatic studies have suggested the presence of more than 12,000 A-to-I editing sites corresponding to more than 1,400 edited mRNAs [42-44]. These sites correspond primarily to non-coding regions of the RNA, typically Alu repeats [42-44]. A hallmark of an A-to-I RNA editing event is an A-to-G transition when comparing genomic and cDNA sequences of the affected gene, since inosine base pairs with cytosine and therefore is replaced by guanosine during reverse transcription [42]. We therefore checked whether tags from our set of unmapped tags for which we could not find any origin could come from A-to-I or C-to-U edited mRNA. For this purpose, we built a matrix containing the transition frequencies between base pairs, by comparing correct tags that match with 100% identity to the genome sequence and their corresponding incorrect variants (unmapped to the genome, and containing one modification by comparison to the corresponding correct tag : these variants could either be due to sequencing errors or to editing). This table shows this matrix, where the rows correspond to the bases in the tags mapped to the genome sequence, and the columns the bases in the corresponding variant tags. We see that the transition frequency from A to G is roughly the same as the transition frequency from G to A. Likewise, the transition fr [file 1471-2105-8-154-S3.pdf]

	A	C	G	T
A	0.950	0.011	0.024	0.015
C	0.015	0.951	0.012	0.022
G	0.023	0.013	0.950	0.014
T	0.014	0.026	0.013	0.947
